# Ischemic Colitis in Buerger’s Disease: Case Presentation and Review

**DOI:** 10.7759/cureus.8303

**Published:** 2020-05-26

**Authors:** Haider A Naqvi, Muhammed Bilal, Shuja Yousuf

**Affiliations:** 1 Internal Medicine, Medstar Union Memorial Hospital, Baltimore, USA; 2 Medicine: Surgery, William Carey University College of Osteopathic Medicine, Hattiesburg, USA; 3 Clinical Gastroenterology & Internal Medicine, Merit Health Rankin & William Carey University College of Osteopathic Medicine, Brandon, USA

**Keywords:** buerger’s disease, ischemic colitis, colonoscopy, gastrointestinal

## Abstract

Buerger’s disease is a type of vasculitis that predominantly affects small to medium arteries and the veins of the upper and lower extremities. Intestinal vessels are rarely involved. This is a case report of a 38-year-old male, smoker, with known Buerger’s disease who was found to have ischemic colitis of the sigmoid colon on biopsy and inferior mesenteric artery occlusion on computed tomography (CT) angiography. Intestinal ischemia is a rare complication in Buerger’s disease. Patients may present with vague abdominal symptoms. Given the very low incidence of intestinal involvement, social history and clinical correlation are of chief importance for early detection. Smoking cessation is paramount, as it is the mainstay treatment of the underlying disease.

## Introduction

Buerger’s disease, also known as thromboangiitis obliterans (TAO), is a condition caused by nonatherosclerotic segmental inflammation of the small and medium arteries of the upper and lower extremities [1]. The occurrence of Buerger’s disease is strongly correlated with smoking [2]. TAO with gastrointestinal complications has been rarely reported with an incidence of 2% and Kyeong Soo Lee et al. have reported that a total of 29 cases have been reported so far in the literature [3]. TAO has historically been known as a male predominant disease because of its association with smoking and age that usually shows during the classical presentation. With an increase in women smokers worldwide, the ratio of affected males to females has changed from 100:1 to 10:1. Worldwide, the disease has been more prevalent in the Middle East and parts of Far East Asia, and the prevalence of the disease has been extremely rare in the US and Europe. Here, we are going to discuss a case of a man with Buerger’s disease who presented with mid-epigastric abdominal pain and underwent colonoscopy and CT angiography and was found to have ischemic colitis with inferior mesenteric artery occlusion.

## Case presentation

**Figure 1:**
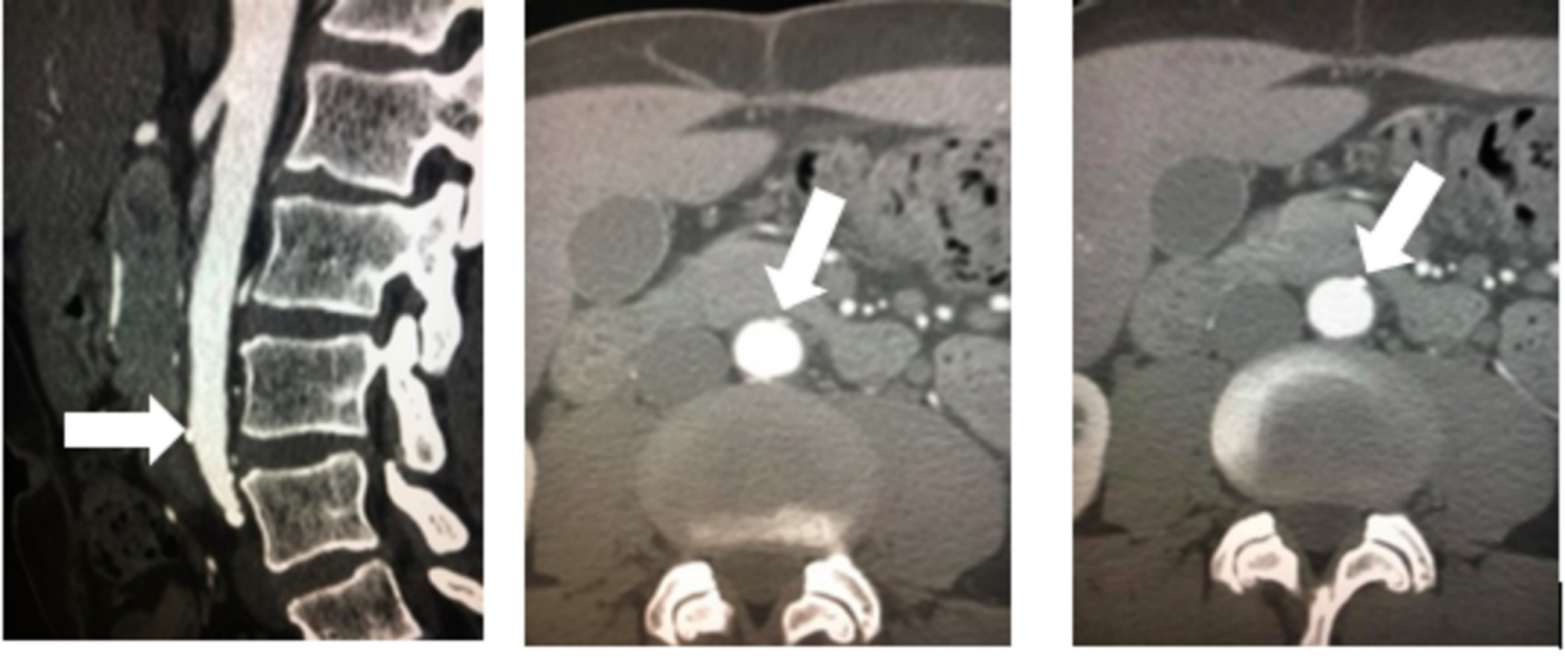
Left to Right: CT angiography showing occlusion of the inferior mesenteric artery, as indicated by the white arrow, at its origin in the sagittal and coronal planes CT: computed tomography

A 38-year-old Caucasian male presented to the clinic for abdominal pain and rectal bleeding. He was diagnosed with Buerger’s disease eight years ago and underwent a left below-knee amputation. He also is a chronic tobacco user, polysubstance abuser, and has a seizure disorder. He reported incidences of intermittent rectal bleeding for at least a week, with increasing frequency and 15-minute postprandial epigastric pain. He denied prior upper endoscopy and nonsteroidal anti-inflammatory drugs (NSAIDs) use. Due to his drug history, he was tested for hepatitis A/B/C and human immunodeficiency virus (HIV), which were both negative. The complete blood count, complete metabolic count, amylase, and lipase were within normal limits; lactic acid was not measured. A CT angiography was performed to rule out mesenteric occlusion of the intestinal vessels due to underlying Buerger’s disease. The CT scan showed that the inferior mesenteric artery appeared to be occluded at its origin (Figure 1).

**Figure 2:**
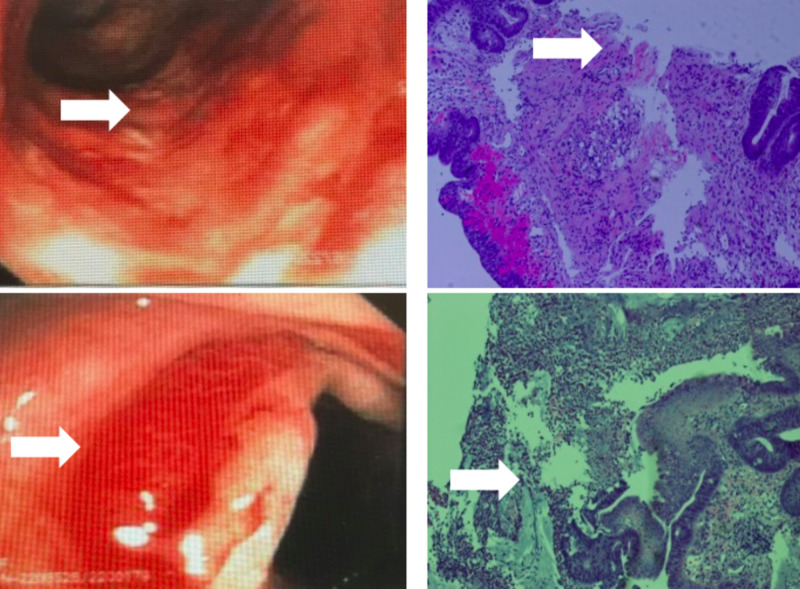
Left column: Top picture shows friable colonic epithelium and area of ischemia (white arrow). Bottom picture shows friable colonic epithelium and active bleeding (white arrow). Right column: Top picture shows the pathology with friable colonic epithelium that is sloughing off (white arrow). Bottom picture shows distorted crypt architecture and membrane (white arrow)

The endoscopy was unremarkable. On colonoscopy, the left colon showed moderate erythema, friable mucosa, and ulceration stretching 12-15 cm at approximately 30 cm from the anal verge (Figure 2). Targeted biopsies were taken from the inflamed colon, with the pathology report showing mucosal ulceration, fibropurulent exudate, and glandular atrophy. These findings are consistent with ischemic colitis.

The patient was strongly advised to discontinue smoking repeatedly after a discussion of the pathology and imaging results. He was also referred to vascular surgery, which he did not attend.

## Discussion

First named in 1908 by Dr. Leo Buerger, Buerger’s disease is a non-atherosclerotic, segmental occlusive vasculitis of the medium and small veins and arteries of the extremities [4]. The prevalence of Buerger’s disease has a worldwide distribution, with the greatest occurrence in countries such as India (45-63%) and Japan and Korea (16-66%) [2]. Due to its strong correlation with smoking, it would be interesting to reevaluate the trend in occurrence with the updated Centers for Disease Control and Prevention (CDC) reports showing a decline and leveled rates of tobacco use since 2008 [5]. The diagnosis of Buerger’s disease is typically made clinically using Shionoya’s clinical criteria, which includes a history of tobacco use, age of onset < 50, occlusion of the infrapopliteal arteries, superficial migratory thrombophlebitis or distal upper limb arterial occlusion, and exclusion of atherosclerosis risk factors other than smoking [6]. Typically larger arteries, such as the mesenteric vessels, are unaffected in Buerger’s disease but, to date, only a total of six cases have been reported to have colonic mesenteric vessel involvement [3]. The mainstay of treatment for Buerger’s disease is the cessation of all tobacco products, including nicotine-containing patches [7-8].

Typically, Buerger’s disease does not affect larger vessels but few cases have been described [9]. Vascular occlusions have been reported to occur in coronary arteries, the central nervous system (CNS), and abdominal viscera. In a literature review done by Hausson et al., since 1955, they found only 23 cases of Buerger’s disease with the involvement of intestinal vessels, with a more recent case review totaling 29; this represents 2% of all reported cases [3-4,9]. The gastrointestinal manifestations of Buerger’s disease tend to affect the celiac, gastric, mesenteric, and colonic vessels with a preference for the smaller branching vessels. Within, the alimentary canal, the small bowel has had the highest predilection of Buerger’s disease. From the total 29 cases to date with intestinal involvement, only six have been known to have colonic involvement. Gastrointestinal involvement of Buerger’s disease is rare, and colonic involvement is as rare as a complication [4].

The exact mechanism of pathophysiology in Buerger’s disease is unknown, but reviewed histopathology from previous studies has shown some insight into the disease process. There are three stages that occur in the spectrum of the disease: acute phase, intermediate phase, and chronic phase [10]. The acute phase is characterized by inflammation through the vessel wall associated with occlusion and the formation of micro-abscesses. The intermediate phase is described as decreased inflammation and organization of the occlusion. Finally, the chronic phase shows recanalization, vascularization of media, and adventitial and perivascular fibrosis. In unusual cases of Buerger’s disease, like in this patient, the diagnosis is based on the histopathological examination showing the acute phase. In our case, the patient presentation (intermittent rectal bleeding with postprandial epigastric pain) plus the colonoscopic findings in the sigmoid colon (erythema, friable mucosa, and ulceration) were highly suggestive of ischemic colitis, which was confirmed on pathological examination. These findings fall into the acute phase of Buerger’s disease that has been described in previous studies.

Because of the lack of a specific laboratory test, a diagnosis of Buerger’s disease is established by clinical assessment. Ancillary laboratory tests are used to rule out other causes of occlusive pathologies. The diagnosis is confirmed with the fulfillment of all five criteria created in 1998 - the diagnostic criteria of Shionoya [11]. This patient had a typical medical history of Buerger’s disease, which was previously diagnosed in 2009. He met all five criteria: age of onset 31, pack per day (PPD) smoking history, left below the knee amputation (BKA) due to arterial occlusion, Raynaud’s phenomenon, and lack of other atherosclerotic manifestations. He was advised to discontinue smoking after the diagnosis of his condition, which he is currently attempting with the initial cut-down of cigarettes.

Known Buerger’s disease patients presenting with new-onset gastrointestinal symptoms need to be evaluated and gastrointestinal involvement needs to be ruled out. Diagnosis needs to be made early to prevent intestinal obstruction and gangrene formation [9]. Patients need to be constantly followed up in the clinic and advised smoking cessation to prevent further involvement of the disease.

## Conclusions

Intestinal involvement in Buerger’s disease is a rare complication. Due to a lack of laboratory studies to identify Buerger’s disease; clinical suspicion, judgment, and history are of primary importance. The differential diagnosis of colonic ischemia should be included in a patient that presents with abdominal pain and a history of Buerger’s disease and careful evaluation and follow-up should be executed. Smoking cessation still remains the primary treatment of the underlying disease process.
